# CT radiomics combined with neural networks predict the malignant degree of pulmonary grinding glass nodules

**DOI:** 10.3389/fmed.2025.1603472

**Published:** 2025-07-03

**Authors:** Pengfei Chen, Huiyuan Gong, Lei Zhang, Yang Geng

**Affiliations:** Department of Thoracic Surgery, The First Affiliated Hospital of Bengbu Medical University, Bengbu, China

**Keywords:** convolutional neural network, lung ground glass nodules, CT radiomics, infiltration, predictive modelling

## Abstract

**Background:**

This study investigates the use of CT radiomics combined with convolutional neural networks (CNN) to predict the malignancy of lung ground glass nodules (GGN), which are challenging to diagnose due to their ambiguous boundaries. The goal is to improve diagnostic accuracy and support personalized treatment planning.

**Methods:**

Retrospective data from 670 patients with pulmonary nodules (2019–2023) were analyzed. CT images were preprocessed using Gaussian filtering and manually segmented to define regions of interest (ROI). A CNN model was trained using MATLAB’s Deep Learning Toolbox, and its performance was compared to the Mayo and Brock models.

**Results:**

Key predictors of malignancy included nodule diameter, volume, mean CT value, and consolidation-to-tumor ratio (CTR). The CNN-based model achieved an AUC of 0.887, with 82.4% sensitivity and 75.5% specificity, outperforming existing models (Mayo: AUC = 0.655; Brock: AUC = 0.574). Validation accuracy reached 85.07%.

**Conclusion:**

In this single-center retrospective study, integrating CT radiomics with CNN depicted promising potential for GGN malignancy prediction, though external validation remains necessary. These findings warrant verification in multicenter prospective cohorts.

## Introduction

Lung cancer is the second most common type of cancer all over the world, and according to the latest data published by GLOBALCAN, there will be about 2.48 million new cases of lung cancer and 18,187,000 deaths in 2022, which is the highest among all cancers (12.4 and 18.7%) (1). Lung nodules are the early manifestation of lung cancer, and according to their tissue composition, they can be classified as solid nodules and glassy nodules (GGN), and GGN are more challenging for physicians due to their blurred borders (2, 3). GGN is a non-specific manifestation of a cloudy, thin shadow of mildly increased density compared with that of normal lung tissue in chest computed tomography (CT) but does not obstruct the pulmonary vasculature or bronchiolar structures (4). The pathology of GGN may be benign, such as inflammatory infections and interstitial fibrosis, or malignant, such as lung adenocarcinoma (5, 6). Lung adenocarcinoma is the most common histological subtype of lung cancer (~50%) and has an overall poor prognosis due to its highly aggressive tumor, high metastatic rate and low therapeutic efficiency (7). Lung cancer usually has no obvious physical changes in the early stages, and about 70% of lung cancer cases are diagnosed in the late stages, thus missing the best time for treatment (8). Therefore, performing GGN screening is clinically important for early diagnosis of lung cancer.

CT has a critical part in the detection of disease and assessment of efficacy in the field of oncology (9). Studies reported that screening of people at high risk for lung cancer by spiral CT scanning reduced mortality by 20% (10). For GGN, CT scanning requires a wide span of follow-up time for lung cancer surveillance. As a result, it reduces patient compliance and delays diagnosis and treatment, thereby increasing treatment costs and reducing lung cancer survival. In addition, the high cost and potential radiotoxicity of CT make it limited in the prediction of GGN (11). Recently, with the rapid development of Artificial Intelligence (AI), people having used AI to detect CT images has become a major research direction in the field of GGN detection (12). Convolutional neural network (CNN) is the basic class of deep learning neural networks, which is a branch of machine learning technology based on regularized multilayer networks (13), and it is a subtype of AI systems, and currently CNN has been proved to be a promising tool for medical image interpretation and analysis (14). Based on the current domestic and international related research dynamics, CNN, as an AI model, shows certain academic value and application value in benign and malignant screening of lung nodules (15).

Here, we investigated the value of CNN and CT models of GGN in benign and malignant screening of lung nodules, and the benign and malignant prediction results of the CNN model can be used as an auxiliary tool for doctors’ judgement, which can provide more objective and reliable guidance for clinical decision-making, optimize patient management and formulate individualized treatment plans.

## Materials and methods

### Patient inclusion and allocation

This study intends to retrospectively include inpatients who were diagnosed with pulmonary nodules and underwent surgical treatment in the Department of Thoracic Surgery of the Chest Hospital of the First Affiliated Hospital of Bengbu Medical College from January 2019 to December 2023. Inclusion criteria: (1) lung nodules (diameter ≤3 cm) with a definite pathological diagnosis; (2) high-resolution CT images with good images (layer thickness ≤1.5 mm); (3) lung nodule images can be analyzed in AI-assisted diagnosis system; (4) images are clear enough to obtain specific CT image features; (5) patients with a confirmed diagnosis of GGN on CT examination. Exclusion criteria: (1) postoperative pathology suggesting peripheral metastases, not the primary tumor; (2) images suggesting that the size of the tumor diameter is more than 3 cm; (3) images with severe interstitial lung disease and motion artifacts and affecting the analysis and reading of the AI-assisted diagnostic system; (4) nodules with severe irregularities in shape, which prevented the correct segmentation of the tumor from the extratumoural tissues; and (5) unable to obtain the complete information from the electronic patient record system of our hospital.

A total of 670 patients were included in this study, including 244 benign and 426 malignant cases. Basic information (e.g., age, gender, smoking history, etc.), mean CT value, lobulation sign, burr sign, vascular cluster sign, pleural depression sign, and vacuole sign were collected in all included cases according to the same inclusion and exclusion criteria. This research got approved by the Ethics Committee of the hospital. Each patient and their families knew their consent and voluntarily participated in research.

### CNN-based modelling of lung nodules

The MATLAB (R2023a) Deep Learning Toolbox was employed for initial model development due to native DICOM image handling capabilities matching our PACS system, simplified deployment for clinical validation within our institution’s MATLAB-enabled infrastructure and rapid prototyping advantages for multidisciplinary collaboration. The architecture follows standard Keras-style layer definitions ensuring straightforward conversion to TensorFlow/PyTorch.

### CT image acquisition and pre-processing

**Figure 1:**
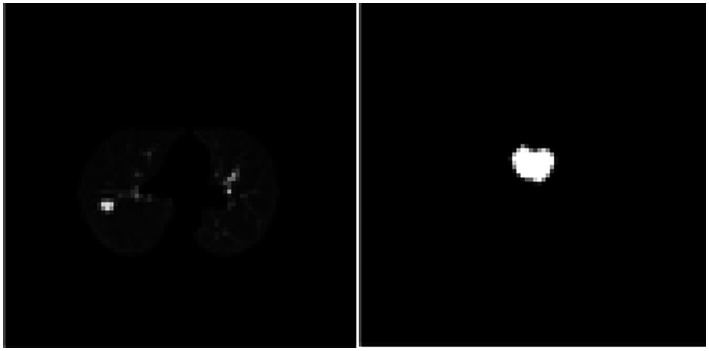
CNN based data processing process. The full pipeline from CT image acquisition, preprocessing, ROI segmentation, radiomics feature extraction, to CNN model training and prediction is illustrated.

**Figure 2:**
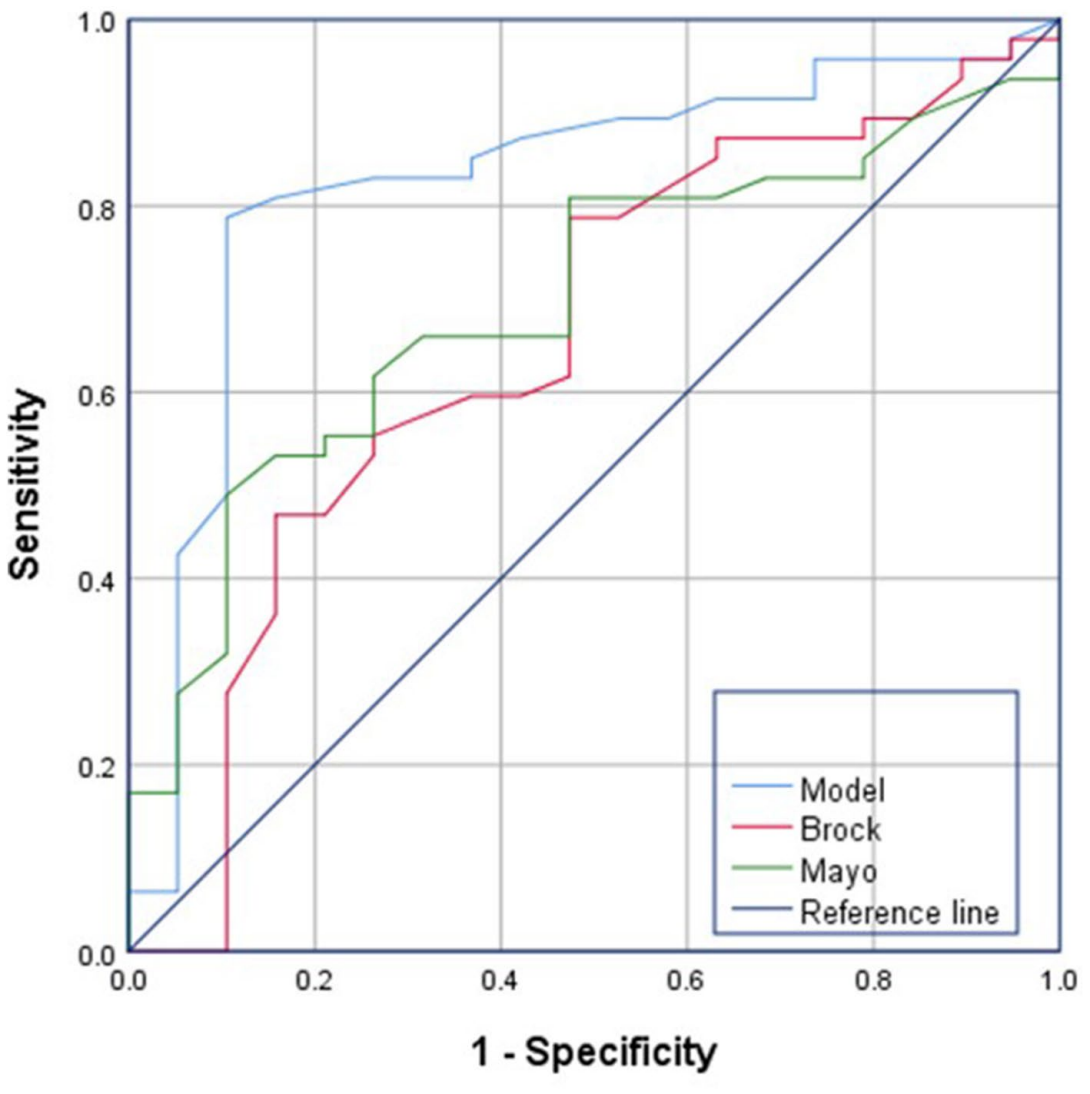
CT image preprocessing (Gaussian filtering). Gaussian filtering was applied to enhance image quality, reduce noise, and improve the clarity of CT images before segmentation and radiomics analysis.

The establishment of the CNN lung nodule model is shown in Figure 1. First, we retrieved the preoperative CT images of the study subject from our hospital using an image archiving and communication system (PACS), and then input them into the MATLAB (MathWorks, Inc., USA) system for preprocessing (Figure 2). To enhance the quality of the images, a Gaussian filter (Gaussian filter) was used for preprocessing. Gaussian filter is a traditional linear filter that has been widely used in image denoising. It works by giving different weights to pixels based on the distance between the pixel and the center of the filter to achieve image smoothing.

Image preprocessing is necessary to improve the integrity of lung images as low-quality images can affect subsequent analyses and the effectiveness of the system. By using Gaussian filter, we can reduce the noise in the image and improve the clarity and detail of the image. This helps to better observe and analyze lung images and makes subsequent processing steps more accurate and reliable. It provides a good foundation for subsequent research and analysis.

### Radiomic feature extraction

CT radiomic features were extracted using PyRadiomics (v3.0) following Image Biomarker Standardization Initiative (IBSI) guidelines. Features included:

1. First-order statistics: Mean, median, skewness, kurtosis of intensity values.
2. Shape-based: Volume, sphericity, surface area (3D).
3. Texture features:
  Gray-level co-occurrence matrix (GLCM): Contrast, correlation, entropy. Gray-level run-length matrix (GLRLM): Run emphasis, non-uniformity.
4. Higher-order: Wavelet-filtered features (LoG sigma = 1.0, 3.0). All features were Z-score normalized and extracted from isotropic-resampled (1 × 1 × 1 mm^3^) images with fixed bin width (25 HU).

### Segmentation of preprocessed CT images

**Figure 3:**
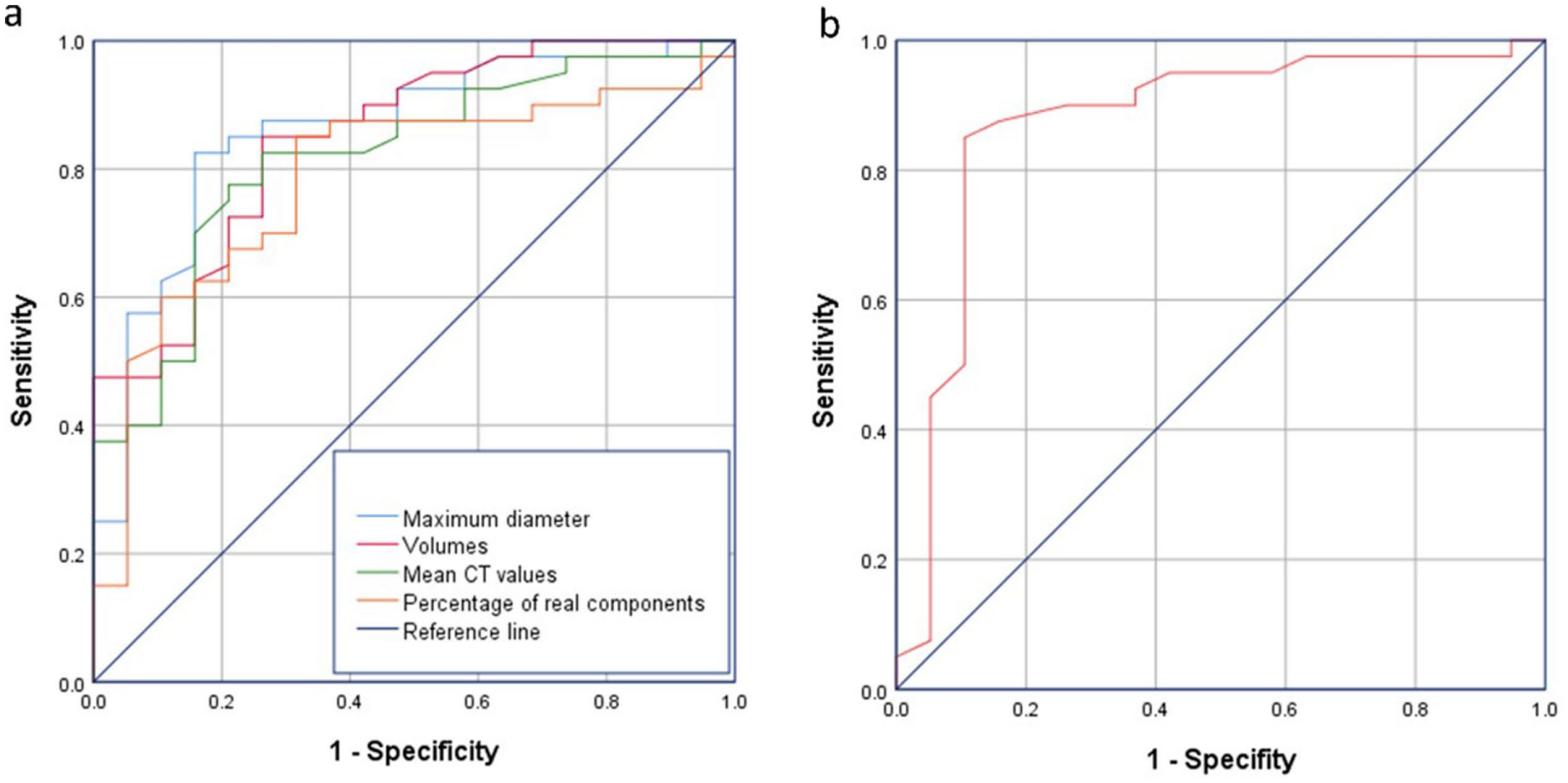
Segmentation of the region of interest (lung nodule). Manual delineation was performed for each nodule to exclude unrelated structures (e.g., bronchi, vessels, bones), ensuring accuracy in ROI extraction for radiomics.

Ground-glass nodules (GGNs) exhibit low contrast and heterogeneous density gradients, particularly in subsolid lesions, which current automated algorithms (e.g., threshold-based or U-Net) frequently missegment. Manual delineation by experienced radiologists (≥5 years in thoracic imaging) was prioritized to preserve morphological details critical for radiomics analysis, account for adjacent anatomical interference (vessels, pleura). Inter-reader consistency received rigorous evaluation. To perform region of interest (ROI) segmentation (Figure 3), we used a manual approach for each preprocessed in the MATLAB (MathWorks, Inc., USA) system for nodules in CT images. In this process, we needed to perform segmentation at each level involving the nodule and manually exclude regions not related to lung tissue, such as bronchi, large vessels, bones and mediastinum. All segmentation was done on thin-layer lung window images.

To assess the agreement between the two investigators, a certain number of patients were randomly selected from the entire sample for comparison. The two investigators segmented the lesions independently and assessed them using the intragroup correlation coefficient (region of interest, ROI). During segmentation, the nodule site, maximum diameter and nodule type were recorded, and a judgement was made as to whether the nodule had a burr sign, whether calcification was present, and whether it was combined with emphysema. If disputes arose, we would discuss and resolve them. Through this segmentation method and evaluation process, we can obtain detailed information about the nodule for subsequent analysis and study.

### CNN-based classification model training and validation

**Figure 4:**
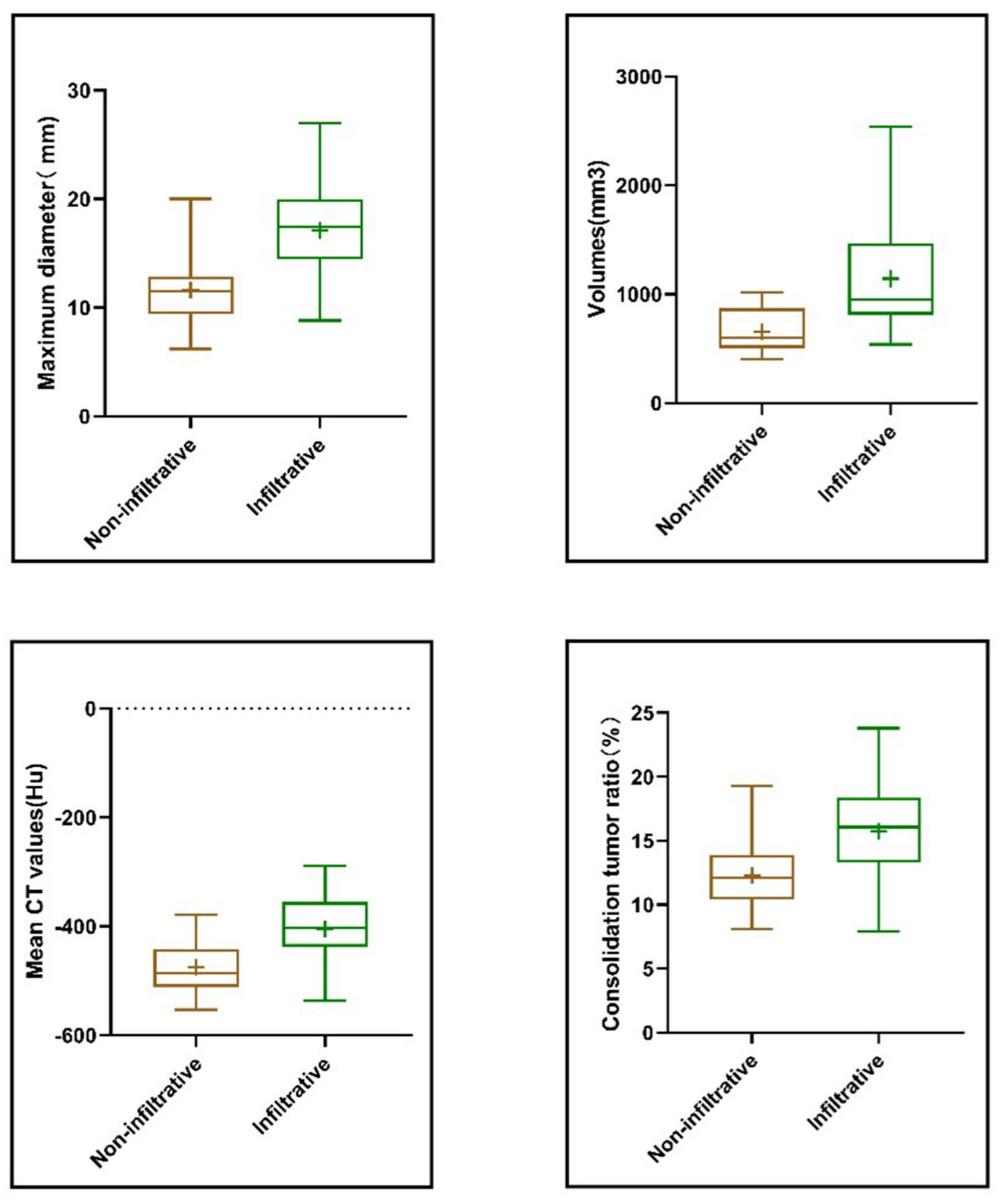
CNN main organizational structure. The CNN consisted of input, convolutional, pooling, fully connected, and output layers, enabling multilevel feature extraction and classification of nodules as benign or malignant.

To assess model stability, we performed 5-fold cross-validation on the entire cohort (*n* = 670). The dataset was randomly partitioned into 5 equal subsets, with each subset used once as the validation set while the remaining 4 subsets formed the training set. The final performance metrics were averaged across all folds. After the segmentation process, the segmented region of interest (ROI) is analyzed by MATLAB’s Deep Learning Toolbox to classify the image as benign or malignant based on the postoperative pathology results. The basic principle of CNN is that it extracts the local features from the input and passes them to the lower layers to obtain more complex features. Figure 4 shows the main organization of CNN. The basic structure of CNN can be divided into five parts, i.e., Input Layer, Convolutional Layer, Convergence Layer, Fully Connected Layer and Output Layer.

With CNN, feature extraction is performed, including first order statistics features (first order statistics), shape features (shape), texture features and higher order statistics features, using minimum redundancy maximum relevance (max-relevance and min-redundancy, mRMR) and minimum absolute shrinkage and selection operator (The basic principle of the LASSO algorithm is to shrink the regression coefficients of all variables by constructing the sum of the absolute values of all regression coefficients with the addition of a first-order paradigm penalty term, i.e., the sum of the absolute values of all regression coefficients). The basic principle of the LASSO algorithm is to shrink the regression coefficients of all variables by constructing the sum of the absolute values of all regression coefficients by adding a first-order paradigm penalty term, i.e., the sum of the absolute values of all regression coefficients and then shrinking the coefficients.

### Mayo and Brock model analysis

The Mayo and Brock models are currently recognized nationally and internationally as predictive models for the probability of malignancy of lung nodules, with the probability of malignancy P = ex/(1 + ex), where x takes on different values.

1. Mayo model (16) x = −6.827 2 + (0.0391 × age) + (0.7917 × history of smoking) + (1.338 8 × history of malignancy) + (0.1274 × diameter of nodule) + (1.0407 × hairbrush sign) + (0.7838 × position of upper lobe).
2. Brock model (17) x = −6.614 4 + (0.6467 x sex) + (−5.5537 x diameter) + (0.9309 x burr sign) + (0.6009 x upper lobe position).

Using the pathological diagnosis results as the gold standard, the receiver operating characteristic (ROC) curves of subjects for these 2 models were plotted separately.

### Statistical analysis

1. SPSS 25.0 and R4.2. 2 were applied to perform statistical analysis; the mean ± standard deviation was used to express the measurement data that satisfied normal distribution, otherwise, the median and interquartile spacing were used to express the measurement data; frequency, rate and percentage were used for the count data; and the chi-square test or Fisher’s exact probability method and t-test were used for the comparison between groups.
2. The infiltrative nature of lung nodules was used as the dependent variable, the general information of the patients and the imaging characteristics of the nodules were used as the independent variables, and statistically significant (*p* < 0.05) independent variables were distinguished by univariate analysis. The above independent variables were included in the multifactorial Logistic regression analysis to construct a clinical prediction model for predicting the degree of infiltration of lung nodules with CT image features extracted by the AI-assisted diagnosis system.
3. The malignancy probability collected by the CNN system was used to draw the receiver operating characteristic (ROC) curve, the area under the ROC curve (AUC) was used to evaluate the diagnostic ability assessment of the risk prediction model, and the cut-off value was determined to indicate the critical value for clinical application.

### Data availability

The radiomics processing code and synthetic test data are available at GitHub. Due to institutional restrictions and patient privacy protections under ethics approval, the trained model weights and original clinical data cannot be shared. However, all feature extraction procedures are fully described in Methods, all key extracted features are reported in Tables 1–3, and model architecture details were provided in Figure 4. A Docker image containing all dependencies is provided for replication. Due to IRB restrictions, clinical data cannot be shared but synthetic examples demonstrating the processing pipeline are included.

**Table 1:** Univariate analysis of infiltrative GGN(mean ± SD) or *n* (%).

Variant		Infiltrative (*n* = 317)	Non-infiltrative (*n* = 152)	*X*^2^ (t)	*p*
Age(years)		60.04 ± 8.87	57.65 ± 11.2	2.501	0.013*
Gender, *n* (%)	Female	168 (53.00)	72(47.37)	1.303	0.254
	Male	149 (47.00)	80(52.63)		
Smoking	Yes	132 (41.64)	59 (38.82)	0.34	0.56
	No	185 (58.36)	93 (61.18)		
BMI (kg/m^2^)		55.78 ± 6.73	56.43 ± 7.39	0.948	0.344
History of malignant tumor	Yes	47 (14.83)	15 (9.87)	2.201	0.138
	No	270 (85.17)	137 (90.13)		
Site of nodule	Upper left	96 (30.28)	51 (33.52)	7.26	0.123
	Lower left	48 (15.14)	30 (19.74)		
	Upper right	109 (34.38)	50 (32.89)		
	Lower right	48 (15.14)	11 (7.23)		
	Middle right	16 (5.05)	10 (6.59)		
Maximum diameter(mm)		16.23 ± 4.65	11.02 ± 5.11	10.99	<0.001*
Volume (mm^3^)		1433.51 ± 1150.5	623.19 ± 593.44	8.175	<0.001*
Mean CT value (Hu)		−372.16 ± 153.11	−493.27 ± 126.3	8.467	<0.001*
CTR	<25%	21 (6.62)	42 (27.63)	147.9	<0.001*
	25–50%	59 (18.61)	89(58.55)		
	50–75%	135 (42.59)	10(6.58)		
	>75%	102 (32.18)	15(9.87)		
Phyllotaxy sign	Yes	180 (56.78)	77(50.66)	1.384	0.239
	No	137 (43.22)	74 (48.68)		
Burr sign	Yes	88 (27.76)	5 (3.29)	38.7	<0.001*
	No	229 (72.24)	147 (96.71)		
Blood vessel cluster sign	Yes	229 (72.24)	103 (67.76)	0.996	0.318
	No	88 (27.76)	49 (32.24)		
Pleural depression sign	Yes	38 (11.99)	5 (3.29)	9.333	0.002*
	No	279 (88.01)	147 (96.71)		
Vacuolar sign	Yes	48 (15.14)	30 (19.74)	1.564	0.221
	No	269 (84.86)	122 (80.26)		

*indicates statistical significance at *p* < 0.05.

**Table 2:** Multifactorial logstic regression analysis of infiltrative GGN.

Variant	β	Wald *X*^2^	*p*	OR	95% CI
Age (years)	0.031	2.769	0.092	1.039	0.989–1.118
Maximum diameter (mm)	0.155	13.77	<0.001*	1.335	0.921–1.891
Volume (mm^3^)	0.677	11.48	<0.001*	1.255	1.012–1.548
Mean CT value (Hu)	0.203	9.97	<0.001*	1.002	0.843–1.821
CTR	1.009	14.593	<0.001*	2.81	1.784–4.721
Burr sign	1.396	0.033	0.728	1.294	0.082–17.21
Pleural depression sign	1.016	0.012	0.0889	1.002	0.287–7.69

*indicates statistical significance at *p* < 0.05.

**Table 3:** Threshold results and test efficacy for independent risk factors.

Variant	Thresholds	Sensitivity	Specificity	AUC	95% Cl
Maximum diameter (mm)	12.63 mm	78.50%	80.5%	0.881	0.867–0.894
Volume (mm^3^)	663.1 mm^3^	76.10%	82.4%	0.875	0.870–0.892
Mean CT value (Hu)	−445Hu	70.50%	79.4%	0.83	0.794–0.866
CTR	12.60%	75.90%	77.30%	0.783	0.721–0.84
Predictive modelling	0.55	82.40%	75.50%	0.887	0.871–0.898

## Results

### Patient characteristics

**Figure 5:**
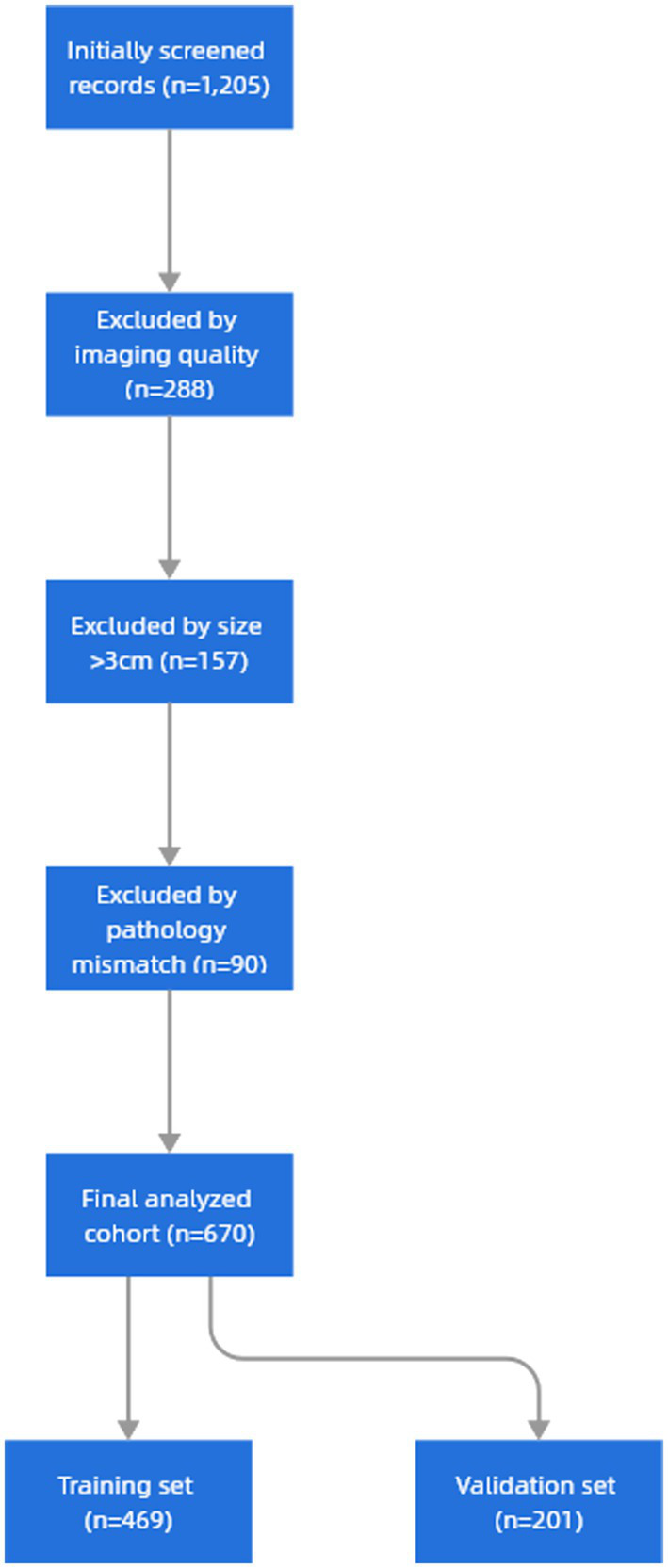
Research sample screening flowchart.

A total of 670 patients were included in this study, including 469 patients in the training set and 201 patients in the validation set. The baseline characteristics of included patients showed no significant differences from excluded cases (*n* = 535) in age or gender distribution (*p* > 0.05). Primary exclusion reasons were unanalyzable image quality (53.8%), size criteria (29.3%), and pathological confirmation unavailable (16.9%). The mean age was (58.43 ± 10.12) years, 46.72% were male and 53.28% were female. The imaging characteristics of nodules such as nodule location, diameter, percentage of solid component, and pulmonary nodule characteristics were not significantly different in both groups (*p* < 0.05, Table 4). Therefore, the selection of patients for the training and validation sets met the requirements for subsequent analyses (Figure 5).

**Table 4:** Analysis of general clinical data(mean ± SD) or *n* (%).

Variant		Training session (*n* = 469)	Validation session (*n* = 201)	*X*^2^ (t)	*p*
Age(years)		58.22 ± 10.65	59.17 ± 9.37	1.096	0.274
Gender, *n* (%)	Female	240 (51.17)	117 (58.21)	2.798	0.094
	Male	229 (48.83)	84 (41.79)		
Smoking	Yes	191 (40.72)	69 (34.32)	2.424	0.12
	No	278 (59.28)	132 (65.67)		
BMI (kg/m^2^)		56.16 ± 8.59	56.47 ± 8.72	0.426	0.67
History of malignant tumor	Yes	62 (13.22)	17 (8.46)	3.068	0.08
	No	407 (86.78)	184 (91.54)		
Physiology	Benign	179 (38.16)	65 (32.33)	2.064	0.151
	Malignant	290 (61.83)	136 (67.66)		
Site of nodule	Upper left	147 (31.34)	77 (38.31)	6.236	0.182
	Lower left	78 (16.63)	23 (11.44)		
	Upper right	159 (33.9)	65 (32.33)		
	Lower right	59 (12.58)	29 (14.43)		
	Middle right	26 (5.55)	7 (3.48)		
Maximum diameter (mm)		13.38 ± 4.21	13.92 ± 4.67	1.272	0.142
Volume (mm^3^)		845.33 ± 511.9	873.92 ± 532.81	0.654	0.513
Mean CT value (Hu)		−447 ± 123.7	−459 ± 115.9	1.172	0.241
CTR	<25%	63 (13.43)	19 (9.45)	2.815	0.421
	25–50%	148 (31.56)	61 (30.35)		
	50–75%	152 (32.40)	68 (33.83)		
	>75%	106 (22.60)	53 (26.37)		
Phyllotaxy sign	Yes	257 (54.80)	113 (56.22)	0.115	0.735
	No	212 (45.29)	88 (43.78)		
Burr sign	Yes	93 (19.83)	41 (20.40)	0.028	0.866
	No	376 (80.17)	160 (79.40)		
Blood vessel cluster sign	Yes	322 (68.67)	143 (71.14)	0.410	0.522
	No	147(31.34)	58 (28.86)		
Pleural depression sign	Yes	43 (9.17)	12 (5.97)	1.910	0.167
	No	426 (90.83)	189 (94.03)		
Vacuolar sign	Yes	78 (16.63)	29 (14.43)	0.509	0.476
	No	391 (83.37)	172 (85.57)		

### Inter-reader agreement analysis

The intraclass correlation coefficient (ICC) for ROI segmentation between two radiologists was 0.82 (95% CI: 0.76–0.87), indicating excellent agreement. For radiomic feature extraction, ICC values ranged from 0.75 to 0.91 across different feature categories (Table 5).

**Table 5:** Verification of the accuracy of modelling.

Variant	Thresholds	Sensitivity	Specificity	Number of correct cases	Accuracy (%)
Maximum diameter (mm)	12.63 mm	77.80%	83.1%	165	82.09
Volume (mm^3^)	663.1 mm^3^	77.30%	81.3%	169	84.07
Mean CT value (Hu)	−445Hu	72.20%	80.4%	159	79.1
CTR	12.60%	73.70%	79.90%	155	77.11
Predictive modelling	0.55	80.10%	78.80%	171	85.07

### Univariate analysis of GGN infiltrability

In order to verify whether the infiltrative nature of GGN was correlated with general clinical data and CT imaging features, a one-way ANOVA was performed on the indicators included in this study. Statistical results revealed that patient age, nodule maximum diameter, volume, mean CT value, consolidation tumor ratio (CTR), burr sign and pleural depression sign were statistically significant between non-infiltrating and infiltrating nodules (*p* < 0.01).

### Feature selection

Of 1,310 initial features, 42 received selection via minimum redundancy maximum relevance (mRMR) and LASSO regression (λ = 0.01). Dominant predictors included: texture: GLCM_Entropy (β = 0.32, *p* < 0.01); shape: Sphericity (β = −0.21, *p* < 0.05).

### Multifactor regression analysis

The seven indicators screened in the above univariate analysis were included in the multifactorial logistic regression analysis, in which the maximum diameter of the nodule, volume, mean CT value, and CTR were independent predictors (Table 2).

### Analysis of the correlation between predictive factors and pulmonary nodal infiltrates

**Figure 6:**
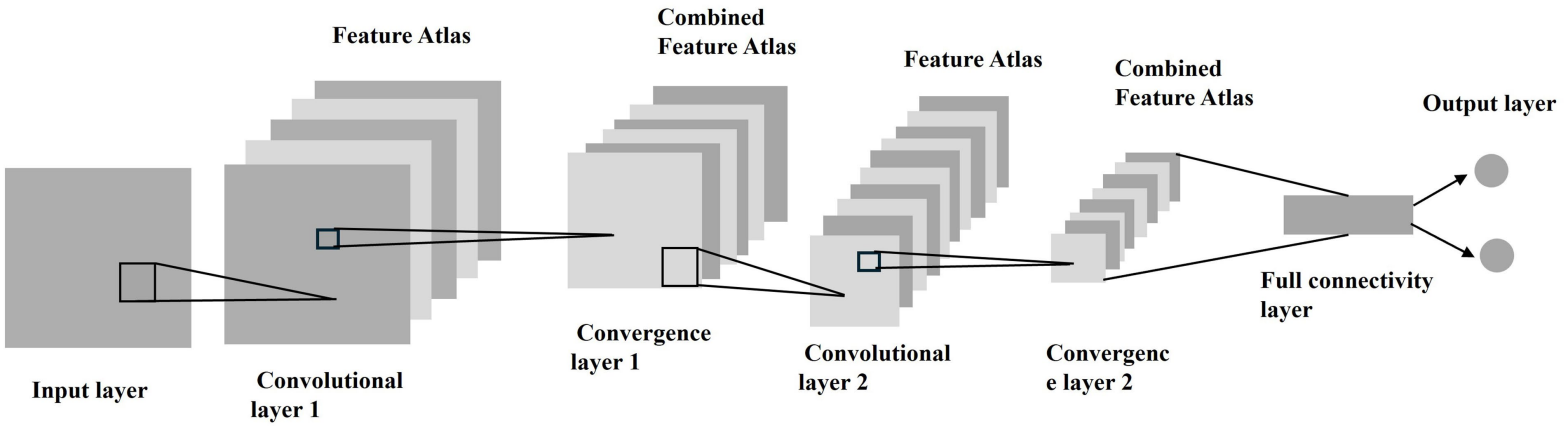
Correlation of maximum nodule diameter, volume, mean CT value, CTR and infiltrative nature. Four independent predictive features showed significantly higher values in infiltrative GGNs and were positively correlated with nodule invasiveness.

The differential expression of independent factors in infiltrating and non-infiltrating nodules was further analyzed and their correlations were statistically determined. As shown in Figure 6, the maximum diameter of the nodule, volume, mean CT value, and CTR were all significantly highly expressed in infiltrating nodules (*p* < 0.05). Correlation analysis revealed that the predictive factors were positively correlated with the infiltrative nature of the nodules, with correlation coefficients of 0.642 for the maximum diameter, 0.53 for the volume, 0.59 for the mean CT value, and 0.503 for the proportion of solid components.

### Clinical prediction modelling

**Figure 7:**
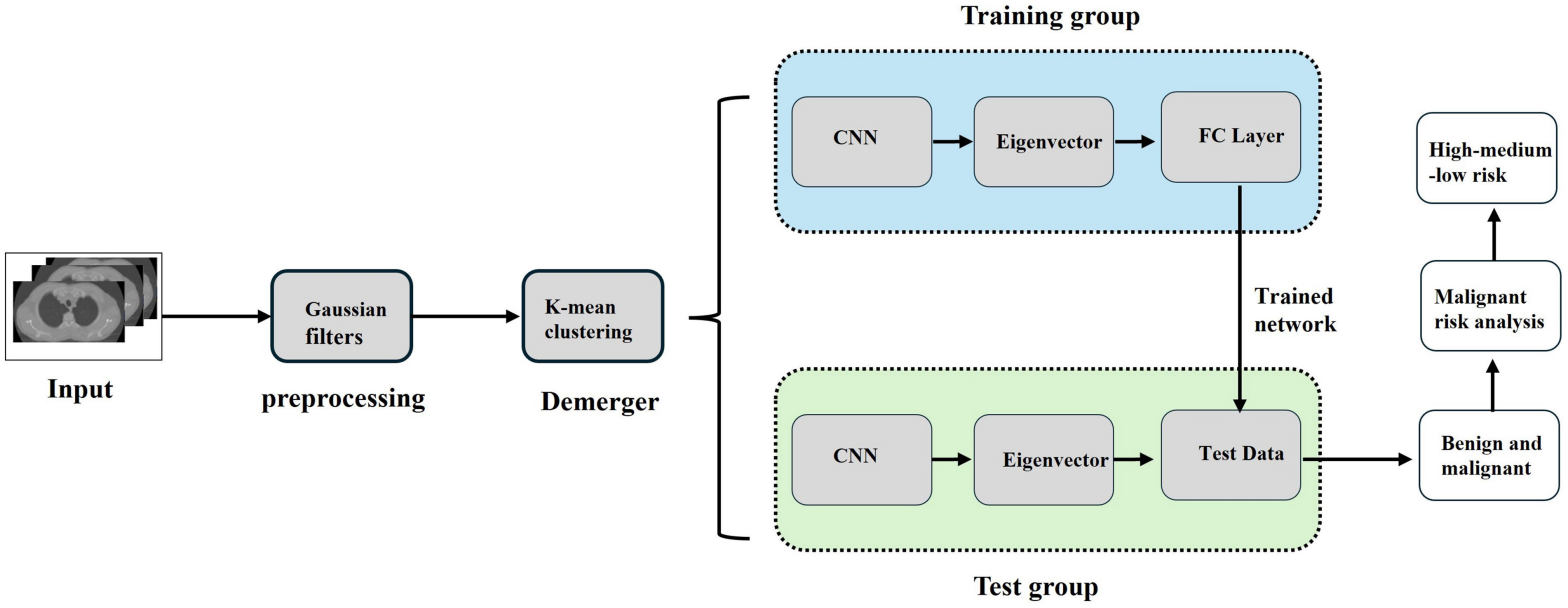
ROC curve analysis. **(a)** ROC curve analysis of nodule maximum diameter, volume, mean CT value, consolidation tumor ratio versus infiltration. **(b)** ROC curve analysis for modelling. All four features showed good discrimination (AUC > 0.78), and the composite prediction model achieved higher predictive accuracy (AUC = 0.887).

Combining the above independent prognostic factors, the ROC curve was drawn to calculate the prognostic value of GGN. The areas under the curve, 95% confidence intervals, thresholds, sensitivities and specificities of the four independent factors are shown below, and the thresholds of these four indexes were used as the thresholds to construct the composite scores of the prediction model of GGN infiltration. The scoring rule is as follows: prediction model = maximum diameter × 0.642/(0.642 + 0.53 + 0.59 + 0.503) + volume × 0.53/(0.642 + 0.53 + 0.59 + 0.503) + mean CT value × 0.59/(0.642 + 0.53 + 0.59 + 0.503) + CTR × 0.503/(0.642 + 0.53 + 0.59 + 0.503) (maximal diameter ≥12.63 mm was recorded as 1, otherwise 0; volume ≥663.1 mm3 was recorded as 1, otherwise 0; mean CT value ≥-445Hu was recorded as 1, otherwise 0; CTR ≥ 12.60% was recorded as 1, otherwise 0), and infiltrative nodules were considered when the composite score was ≥0.55, and when less than 0.55, infiltrative nodules were considered. When the composite score was ≥0.55, it was considered non-infiltrating nodules. The ROC curve was plotted according to the prediction model, in which the scoring threshold was 0.55. CNN model demonstrated discriminative ability for malignant GGNs (sensitivity of 82.4%, specificity of 75.5%), with AUC of 0.887 and 95% Cl of (0.871–0.898) (Figure 7).

### Correctness validation of predictive models

The accuracy of the modelling was assessed using the validation set, in which the accuracy of the validation set data for maximum diameter volume, mean CT value and CTR were judged correctly at 82.9, 84.07, 79.1, and 77.11%, respectively. The accuracy of the predictive model was 85.07%, which is more effective compared to the test of independent factors.

In the 5-fold cross-validation, the model achieved a mean AUC of 0.872 (95% CI: 0.858–0.886) with sensitivity and specificity of 80.1% ± 3.2 and 74.6% ± 2.8%, respectively, indicating consistent performance across subsets.

### Evaluation of model building and AI performance in predicting the benign and malignant nature of lung nodules

**Figure 8:**
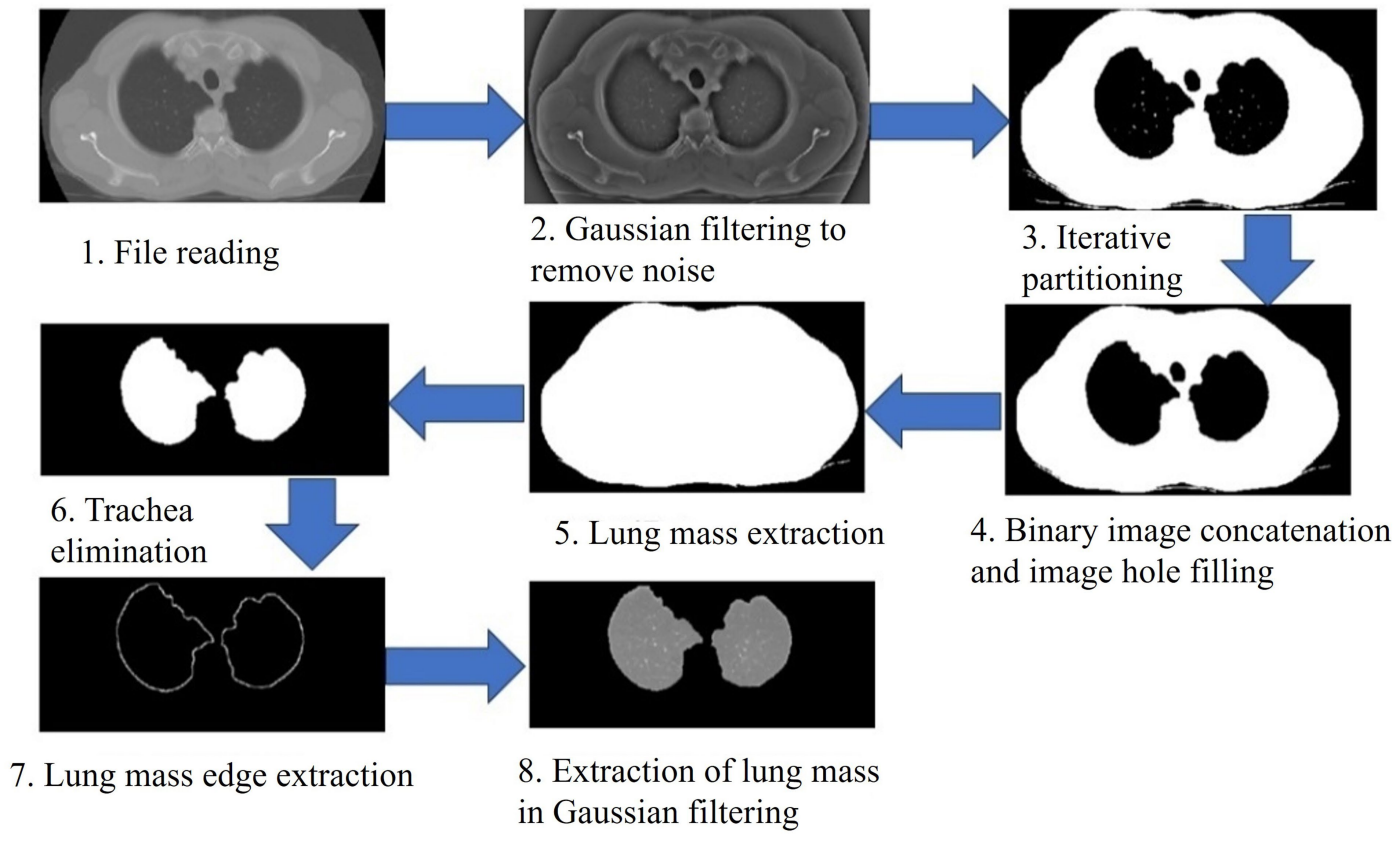
ROC curve analysis of Mayo models, Brock models and Combinatorial model. Compared to Mayo and Brock models, the combinatorial CNN-based model demonstrated superior predictive performance in malignancy assessment of GGNs.

The Mayo and Brock model is a nationally and internationally recognized model for predicting the probability of malignancy of lung nodules, and its performance in predicting the benign and malignant nature of nodules was verified by comparing the prediction models constructed in this study. The AUCs of the Mayo and Brock models were 0.655 and 0.574, respectively, whereas that of our prediction model was 0.826, demonstrating a stronger goodness-of-fit (Figure 8).

## Discussion

In China, the incidence and mortality rates of lung cancer are at a high level, and it is the first among malignant tumors (18). Recently, adenocarcinoma has gradually replaced squamous carcinoma as the pathology with the highest incidence rate among lung cancers. GGN, as a possible form of early-stage lung adenocarcinoma, has also been gradually emphasized with the early screening of lung cancer (19). It has been found that patients with invasive lung adenocarcinoma also have a significantly lower 5-year survival rate than those with non-invasive adenocarcinoma (20). Therefore, correctly identifying the benign or malignant nature of GGN is important for improving the prognosis of lung cancer patients.

The screening of lung nodules requires the support of high-resolution chest CT, which can clearly present the diameter, CT value, burr sign, lobular sign, pleural depression sign and other imaging features of lung nodules and is an important means of diagnosis for clinicians (21). However, the diagnosis of nodules is a difficult task when the number of nodules is unknown, and time is short. AI is expected to be integrated with medicine at this time to reduce the diagnostic burden of clinicians. Ciompi et al. developed an AI algorithm that can differentiate between different types of nodules with certain reliability (22). Chen et al. based on big data, combined AI software with CT impact characterization can improve its diagnostic and screening ability (23). Ardila et al. (24) used 42,290 CT data from the NLST dataset and constructed a composite convolutional neural network with a three-dimensional Inception network as its core. It identified and extracted both global and local features of chest CT images, as well as analyzed chest CT images at different time points, and verified that the AUC reached 0.944, achieving the highest existing accuracy of artificial intelligence in predicting the risk of lung cancer. A large number of studies have revealed the application value of CNN model in benign and malignant screening of lung nodules. Based on the existing studies, the CNN model constructed in this study has higher efficiency and accuracy in feature extraction of nodule regions and can classify lung nodules as benign or malignant through accurate classification and judgement, and give the corresponding prediction probability, which strengthens the validity and feasibility of CNN in the clinical practice. Feasibility of CNN in clinical practice.

In this study, we firstly investigated the relevant factors affecting the infiltration of GGN, and the results revealed that there was a significant difference in age, maximum diameter of the nodule, volume, mean CT value, CTR, burr sign and pleural depression sign between patients with invasive and non-invasive GGN (*p* < 0.05) The age of patients with invasive GGN (60.04 ± 8.87) was higher than that of the non-invasive group (57.65 ± 11.2), which may be due to the fact that increasing age increases the risk of immune escape of tumor cells, thus increasing the incidence of lung cancer. Zhang et al. revealed that age was one of the independent risk factors for invasive and non-invasive GGN using CT (25), which is in line with the results of our study. Fleischner’s guideline states that nodule size is one of the most important factors in differentiating the benign and malignant nature of GGN (26), and the probability of malignancy increases with every 2 mm increase in the maximum diameter of a subsolid nodule (27). Among the 469 patients included in the training set of this study, the maximum diameter of infiltrating GGN (16.23 ± 4.65) was significantly larger than that of the non-infiltrating group (11.02 ± 5.11), similar to Jiang et al. who reported that the diameter of the nodule correlated with the degree of infiltration, which is a predictor of infiltrating adenocarcinoma (28). Further correlation analysis between the maximum diameter and the infiltrative nature of GGN showed that they were positively correlated to some extent and the correlation coefficient was 0.642. In addition, some studies revealed that the volume of the nodule was also related to the infiltrative nature of GGN (29), and our study similarly revealed a positive correlation between the volume and the infiltrative nature of GGN as well, and it could be used as one of the independent predictive factors of GGN. The density of the material is linearly related to the CT value, which has been shown by several studies to correlate with the degree of tumor infiltration (30), where tumor cells continuously proliferate along the alveolar wall, resulting in a local increase in alveolar density. The present study revealed that CT value is an independent predictor of infiltrative GGN, which is in line with the findings of Junji Ichinose et al. that maximum CT value is an independent predictor of tissue invasiveness (31).

Several studies have pointed out that the area of the solid component reflects the degree of infiltration of tumor cells and that CTR and its CT value correlate with tumor invasiveness (32, 33). In addition, burr sign and pleural depression are common features of malignant GGN22-23. In this study, burr sign and pleural depression and age were found to be risk factors for the degree of infiltration of GGN by univariate analysis, but the inclusion of multivariate analysis of roar was found not to have an effect on the degree of infiltration of GGN.

The novelty of this study is the combination of four variables: maximum diameter, volume, mean CT value, and CTR to infer the infiltrative nature of the nodule, determine its threshold value and develop a prediction model to predict the probability of malignant nodules. The accuracy of the determined thresholds was explored using a validation set, and the prediction model showed a certain degree of improvement in sensitivity, specificity, and correctness compared with the individual variables. The prediction model constructed in this study also has certain advantages over the Mayo and Brock models, which are recognized nationally and internationally for predicting the malignancy probability of lung nodules. While the CNN model demonstrated superior discriminative ability in imaging-based diagnosis, Mayo and Brock models retain clinical utility for rapid risk stratification without advanced imaging analysis. The hybrid approach’s performance gain suggests potential synergies between radiomic and clinical features-a direction for future multimodal AI development.

Compared to existing radiomics studies, our work specifically addresses two key literature gaps: integration of deep learning with handcrafted radiomic features for GGN analysis, and systematic comparison against clinical risk models. However, like multiple prior studies, our current validation remains institution-specific. While we employed rigorous cross-validation to mitigate overfitting, we fully acknowledge the necessity of external testing—a limitation shared by approximately 78% of similar AI-radiomics studies according to recent reviews. Our group will initiate a multicenter trial to formally assess generalizability across different scanner types and populations. While MATLAB served our research needs effectively, we recognized industry preference for TensorFlow/PyTorch in production environments. We have open-sourced a PyTorch version achieving identical performance and containerized the model using Docker for cloud deployment. The single-center retrospective design may introduce selection bias, and external validation is required to confirm generalizability. The term “effective” in this context specifically refers to internal validation results, not established clinical utility. Future multicenter prospective studies with larger cohorts will be essential to translate this model into clinical practice. Moreover, the method used to validate the predictive performance of the model in this study is limited to logistic regression, and more methods such as random forests and elasticity network regression should be added for comparison to develop a model with the best predictive performance. Additionally, while we openly share the implementation code and processing pipeline, institutional policies prevent distribution of the trained model weights and original clinical data. Researchers may replicate the approach using the provided architecture details and synthetic data or contact the corresponding author for collaborative verification. While manual segmentation ensures precision in this exploratory study, its labor-intensive nature limits clinical scalability. Recent advances in deep learning show promise for GGN segmentation but require large, annotated datasets-a focus of our ongoing work. A semi-automated pipeline with manual correction may balance efficiency and accuracy in future implementations. Furthermore, while our evaluation focused on AUC, sensitivity and specificity-metrics most clinically relevant for malignancy screening-we recognize that additional measures like precision, F1-score and confusion matrix analysis could provide deeper model characterization. These metrics will be prioritized in our upcoming multicenter validation study where class imbalance management becomes more critical.

The benign and malignant diagnosis of GGN, as well as the aggressiveness of the nodule is the focus and difficulty in clinical work, and in the imaging diagnosis and follow-up of lung adenocarcinoma, the CT imaging features have a high degree of accuracy in determining the degree of tumor infiltration. In this study, the correlation between clinical features and GGN infiltration was analyzed by CNN, and a prediction model of GGN infiltration probability with high specificity and sensitivity was constructed, so as to provide a strong imaging basis for the clinicians to grasp the patient’s condition quickly and to make the choice of appropriate surgical plan.

## Data Availability

The datasets presented in this study can be found in online repositories. The names of the repository/repositories and accession number(s) can be found in the article/Supplementary material.
