# Supplementary material for: CT radiomics combined with neural networks predict the malignant degree of pulmonary grinding glass nodules
Source: Front Med (Lausanne). 2025 Jul 3;12:1603472. doi: 10.3389/fmed.2025.1603472 (PMC12267015; doi:10.3389/fmed.2025.1603472)
Supplement: Supplementary file 1 [file Table_1.docx]

Supplementary Table 1. CNN architecture and training specifications

| **Component** | **Parameters** |
| --- | --- |
| Input layer | 512×512×1 (H×W×C) |
| Convolutional blocks | 4 blocks, each with:  Conv2D (kernel=3×3, filters=32/64/128/256)  BatchNorm  ReLU  MaxPooling (2×2) |
| Fully connected | 2 dense layers (512/128 units) |
| Output | Sigmoid (binary classification) |
| Training | Epochs=100, batch=32, lr=0.001 |
| Optimization | Adam (β1=0.9, β2=0.999) |
| Regularization | Dropout=0.5, L2=0.001 |
